# Body surface area at birth and later risk for gestational diabetes mellitus among primiparous women

**DOI:** 10.1007/s00592-018-1256-2

**Published:** 2018-11-14

**Authors:** Senja Masalin, Kristiina Rönö, Hannu Kautiainen, Mika Gissler, Johan G. Eriksson, Merja K. Laine

**Affiliations:** 1 Department of General Practice and Primary Health Care, University of Helsinki and Helsinki University Hospital, Helsinki, Finland; 2 Department of Obstetrics and Gynecology, University of Helsinki and Helsinki University Hospital, Helsinki, Finland; 3 Folkhälsan Research Center, Helsinki, Finland; 4 Primary Health Care Unit, Kuopio University Hospital, Kuopio, Finland; 5 Information Department, National Institute for Health and Welfare, Helsinki, Finland; 6 Department of Neurobiology, Care Sciences and Society, Karolinska Institute, Stockholm, Sweden; 7 Vantaa Health Center, Vantaa, Finland; 8 Department of Chronic Disease Prevention, National Institute for Health and Welfare, Helsinki, Finland

**Keywords:** Body surface area at birth, Birth length, Birth weight, Gestational diabetes mellitus

## Abstract

**Aims:**

To assess the relationship between body surface area (BSA) at birth and future risk for gestational diabetes mellitus (GDM).

**Methods:**

This is an observational cohort study from Vantaa, Finland. The cohort included 1548 Finnish primiparous women, aged 15–28 years, without pre-existing diabetes, who gave birth 2009–2015. All women were born full-term and had complete information about their birth weight and length, from the Finnish Medical Birth Register. Additional data for the study were provided by individual patient health records and Statistics Finland. Study participants were divided into five levels (I–V) according to BSA at birth, based on normal distribution.

**Results:**

There was an inverse association between BSA at birth and risk for GDM (*p* = 0.015 for linearity, after adjustments for age, educational attainment, pre-pregnancy BMI and smoking). The odds ratio (OR) for GDM in level V, with the largest BSA at birth, compared with level I, with the smallest BSA at birth, was 0.43 [95% confidence interval (CI) 0.22–0.83]; adjusted for age, educational attainment, pre-pregnancy body mass index and smoking. The OR for GDM was 0.8 (95% CI 0.68–0.95, *p* = 0.009) for each one standard deviation increase in BSA at birth, adjusted for the same confounders. BSA at birth correlated with adult anthropometry: correlation coefficients were *r* = 0.16 (95% CI 0.11–0.21) for weight, *r* = 0.31 (95% CI 0.26–0.35) for height, and *r* = 0.06 (95% CI 0.01–0.11) for BMI.

**Conclusions:**

Body surface area at birth is inversely associated with future risk for GDM in primiparous women.

## Introduction

Gestational diabetes mellitus (GDM) is a common pregnancy disorder and has long been defined as any degree of abnormal glucose metabolism, first detected or with first onset, during pregnancy [1]. The prevalence of GDM has been rising over the last decade [2, 3], making GDM a serious global health issue today.

Gestational diabetes mellitus has adverse short- and long-term effects on the pregnant woman and her child, both during pregnancy and later in life [4, 5], with maternal obesity augmenting the adverse effects on neonatal outcomes [6]. Some well-known risk factors for GDM include increase in maternal age, genetic predisposition for diabetes mellitus, non-Caucasian ethnicity, as well as maternal obesity [7], possibly also lifestyle and dietary related factors, parental smoking and an early age at menarche [7, 8]. A recent study emphasizes the importance of recognizing GDM as a heterogenic disorder with also non-obese women being affected—indicating the importance of a large number of risk factors [9]. Further, studies have shown that maternal stature alters glucose metabolism and that short women are at increased risk for GDM [10].

According to the Developmental Origins of Health and Disease (DOHaD) hypothesis prenatal environmental conditions might have long-lasting effects and affect an individual´s morbidity in adulthood [11], influencing for example the risk for cardiovascular and metabolic disturbances [12]. In several previous studies, a negative linear association between birth weight and risk for type 2 diabetes (T2D) has been observed [13, 14], although some findings indicating a U-shaped relationship between birth weight and risk of T2D exist [15]. The relationship between birth weight and risk for GDM has also been studied. The results have been conflicting as both studies indicating an inverse association [16–18], as well as a U-shaped relationship have been reported [19–21]. However, birth weight is a very crude measurement of body size at birth. Interestingly, only one recent study has assessed the relationship between maternal birth size using ponderal index (PI) as a measurement of body size at birth and risk for GDM, showing an inverse association [22].

In 2016, we initiated a long-term follow-up study in the city of Vantaa, Finland, to assess both short- and long-term consequences of glucose metabolism on pregnant women and their offspring’s health. The relationship between body size at birth and future risk for GDM has previously been studied using primarily birth weight as a measure of body size at birth. Body surface area (BSA) is an anthropometric measurement used to make a more accurate evaluation of metabolic mass and body size. The aim of this study is to evaluate how BSA at birth affects the future risk for GDM.

## Materials and methods

### Study population

This is an observational cohort study from the city of Vantaa, which is part of the Helsinki metropolitan area and the fourth biggest city in Finland, with around 220,000 inhabitants. During a 7-year follow-up, between January 1st 2009, and December 31st 2015, 13,530 women from Vantaa gave birth. Of these, 1548 primiparous women were Finnish (born in Finland with Finnish or Swedish as native language), without previously diagnosed diabetes mellitus, born at term after the year of 1987 (when the Finnish Medical Birth Register was founded), and aged 15–28 years during the follow-up period. All these women had complete data from the birth register and thereby formed the study cohort.

Data on maternal-fetal characteristics and pregnancy outcomes were obtained from the Finnish Medical Birth Register, which is administrated by the National Institute for Health and Welfare in Finland and receives the information about all live and stillbirths, from 22 gestational weeks or an offspring weight of at least 500 g onwards, from all Finnish maternity hospitals (http://www.thl.fi/en/statistics/parturients). The register started to collect information on deliveries on a nationwide basis from the year 1987. Therefore, only women born 1987 or later were included in this study. The following information about the women was obtained from this source: their own birth weight and birth length, pre-gestational weight in adulthood, adult height, previous pregnancies (miscarriages, induced abortions or ectopic pregnancies), infertility treatments, information about current pregnancies and deliveries, smoking during pregnancy, and hospitalization due to hypertension during pregnancy. Information about GDM, height, and weight was further completed by information from Vantaa Health care patient records.

Since 2008, the nationwide Finnish Current Care Guidelines for GDM recommends screening of all pregnant women during their first pregnancy for GDM using a standard 2-h 75-g oral glucose tolerance test (OGTT), except in women at low risk; that is women with a BMI 18.5–25 kg/m^2^, aged under 25 years and with no family history of diabetes mellitus [23]. The screening is routinely performed between gestational weeks 24 and 28, except in women at high risk; that is women with a pre-pregnancy BMI > 35 kg/m^2^, glucosuria in early pregnancy, family history of diabetes, polycystic ovarian syndrome (PCOS), or use of oral corticosteroids. In these high-risk patients GDM screening is performed earlier in pregnancy between gestational weeks 12 and 16, and if OGTT is normal, it will be repeated between gestational weeks 24 and 28 [23].

GDM was defined according to the Finnish Current Care Guidelines for GDM as one or more pathological glucose values in a standard 2-h 75-g OGTT. The diagnostic thresholds were: fasting plasma glucose ≥ 5.3 mmol/L, 1-h glucose ≥ 10.0 mmol/L, and 2-h glucose ≥ 8.6 mmol/L [23].

Educational attainment was defined according to years of schooling, as obtained from Statistics Finland [Official Statistics of Finland (OSF): http://www.stat.fi/til/vkour/index_en.html].

Body size, taking both weight and height into account, was calculated as BSA, using mathematical formulas. BSA is an anthropometric measurement of interest, in order to make a more accurate evaluation of metabolic mass and body size as a whole. In our study, BSA at birth was calculated according to the *Meban*-BSA formula [24], which in 2008, as evaluated by Ahn et al., has been proved to be the most accurate formula to calculate infant-BSA [25]. Adult pre-pregnancy BSA was calculated according to the commonly used *Mosteller*-BSA formula in adults [26].

#### Statistical analyses

Data are presented as means with standard deviations (SD) or range, or as counts with percentages. The study population was divided into five levels according to birth BSA levels, based on normal distribution, and corresponding to grades containing 12.5, 25, 25, 25, and 12.5% of the total distribution. Cut-offs for birth BSA levels were: 2011 cm^2^ for level I, 2012–2170 cm^2^ for level II, 2171–2291 cm^2^ for level III, 2292–2450 cm^2^ for level IV, and ≥ 2451 cm^2^ for level V. Statistical significances for the unadjusted hypothesis of linearity across categories of birth BSA levels were investigated by the Cochran–Armitage test for trend and analysis of variance (ANOVA) with an appropriate contrast. Adjusted hypotheses of linearity (orthogonal polynomial) and the association between birth BSA and GDM prevalence were evaluated using logistic models. Models included age, educational attainment, pre-pregnancy body mass index, and smoking as covariates. By using 5-knot-restricted cubic spline regression, a possible nonlinear relationship between prevalence of GDM or maternal birth BSA and PI was assessed. The length of the distribution of knots was located at the 5th, 27.5th, 50th, 72.5th, and 95th percentiles. The relationship between birth BSA and pre-pregnancy weight, height and BMI was assessed using correlation coefficients calculated by the Pearson method. Correlation coefficients less than 0.2 were considered very weak, between 0.2 and 0.4 weak, between 0.4 and 0.6 moderate, between 0.6 and 0.8 strong, and above 0.8 very strong [27]. The normality of the variables was tested using the Shapiro–Wilk W test. Statistical significance was set at *p* < 0.05. Stata 15.0 (StataCorp LP; College Station, TX, USA) statistical package was used for the analyses.

## Results

### Characteristics of the study participants

**Fig. 1:**
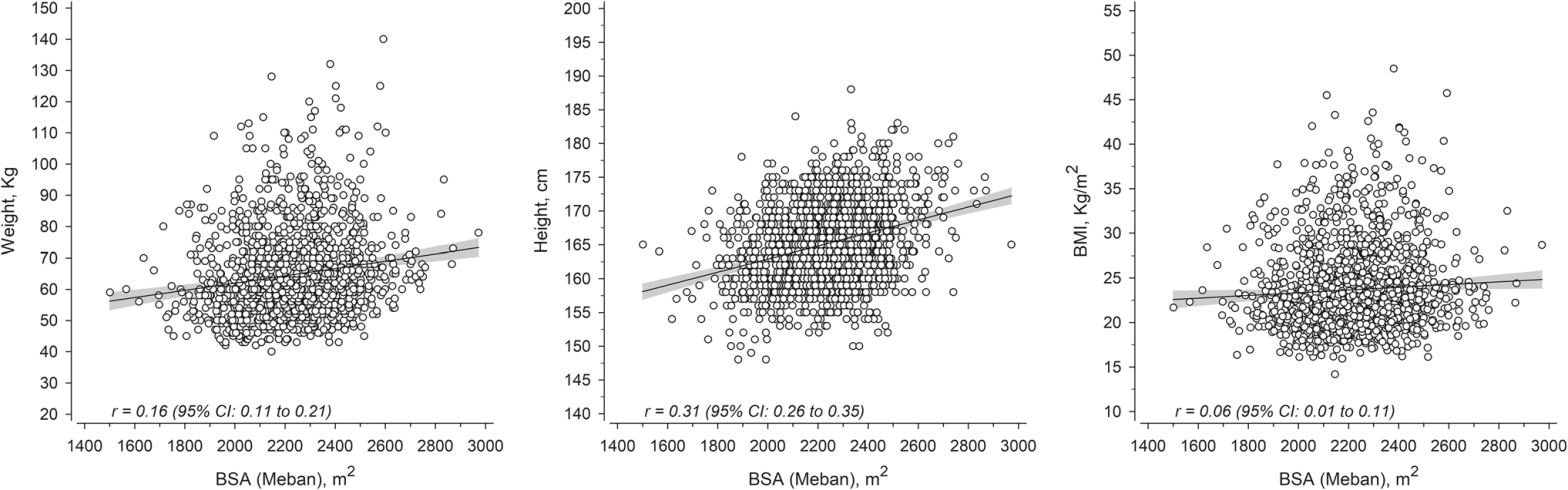
Relationship between maternal body surface area (BSA) at birth and maternal anthropometry. The relationship between maternal BSA at birth, and maternal adult pre-pregnancy weight, height and BMI shown on scatter plots. *BSA* body surface area, *BMI* body mass index

The mean age of the study participants was 22.4 (SD 2.7) years. Baseline characteristics of the 1548 women, divided into five levels, according to body surface area (BSA) at birth is shown in Table 1. Mean BSA at birth was 2231 cm^2^ (SD 191), mean ponderal index was 28.2 kg/m^3^ (SD 2.5), mean birth weight was 3520 g (SD 472), and mean birth length was 49.9 cm (SD 2.0), respectively. BSA levels at birth showed a positive association with adult pre-pregnancy weight, height and BSA (all *p* < 0.001 for linearity), as well as with pre-pregnancy BMI (*p* = 0.004). There was no significant difference between the different BSA groups at birth and prevalence of hypertensive disorders during pregnancy (*p* = 0.20). Evaluation of the relationship between BSA at birth, and adult pre-pregnancy weight, height and pre-pregnancy BMI as continuous values showed an overall weak correlation, with the strongest relationship between BSA at birth and adult height. The correlation coefficients were *r* = 0.16 [95% confidence interval (CI) 0.11–0.21] for pre-pregnancy weight, *r* = 0.31 (95% CI 0.26–0.35) for adult height, and *r* = 0.06 (95% CI 0.01–0.11) for pre-pregnancy BMI (Fig. 1).

**Table 1:** Baseline characteristics of 1548 Finnish primiparous women according to body surface area (BSA) at birth

	BSA level at birth					*p* value for linearity
	I *N* = 183	II *N* = 383	III *N* = 404	IV *N* = 382	V *N* = 186	
Maternal birth characteristics						
BSA (cm^2^), mean (range)	1910 (1501–2011)	2097 (2012–2170)	2232 (2171–2291)	2362 (2292–2450)	2547 (2451–2972)	–
Birth length (cm), mean (SD)	47.1 (1.4)	48.8 (1.2)	50.0 (1.1)	51.1 (1.2)	52.5 (1.3)	< 0.001
Birth weight (g), mean (SD)	2755 (203)	3185 (117)	3508 (97)	3836 (122)	4321 (266)	< 0.001
Ponderal index (kg/m^3^), mean (SD)	26.4 (2.2)	27.5 (2.4)	28.1 (2.3)	28.9 (2.2)	30.0 (2.4)	< 0.001
Maternal adult characteristics						
Age (years), mean (SD)	22.3 (2.6)	22.3 (2.8)	22.2 (2.6)	22.7 (2.8)	22.7 (2.7)	0.024
Cohabiting, *n* (%)	125 (68)	270 (71)	285 (71)	266 (68)	132 (71)	0.98
Smoking^a^, *n* (%)	66 (36)	127 (33)	152 (38)	127 (32)	55 (30)	0.23
Years of education, mean (SD)	11.3 (1.9)	11.2 (2.1)	11.6 (2.1)	11.8 (2.2)	11.9 (2.1)	< 0.001
Height (cm), mean (SD)	162 (6)	164 (5)	165 (5)	166 (6)	169 (6)	< 0.001
Pre-pregnancy weight (kg), mean (SD)	60.6 (12.2)	63.4 (14.0)	64.2 (12.6)	66.5 (15.0)	68.6 (14.1)	< 0.001
Pre-pregnancy BMI (kg/m^2^), mean (SD)	23.0 (4.2)	23.6 (4.9)	23.5 (4.5)	24.2 (5.2)	24.1 (4.7)	0.005
BSA (m^2^), mean (SD)	1.64 (0.18)	1.69 (0.19)	1.71 (0.17)	1.74 (0.21)	1.78 (0.19)	< 0.001
Number of fetuses > 1	0 (0)	2 (1)	1 (1)	3 (1)	1 (1)	0.36
Previous pregnancies	49 (27)	75 (20)	70 (17)	72 (18)	33 (18)	0.043
Fertility treatment, *n* (%)	3 (2)	10 (3)	6 (1)	7 (2)	4 (4)	0.84
Hypertensive disorders^b^, *n* (%)	16 (9)	24 (6)	16 (4)	21 (5)	11 (6)	0.20

*BMI* body mass index, *BSA* body surface area, *SD* standard deviation

^a^Included those who quitted smoking during pregnancy

^b^Hospitalization due to hypertension during pregnancy (including ICD-10 codes O10, O13, and O14)

### BSA at birth and prevalence of GDM

**Fig. 2:**
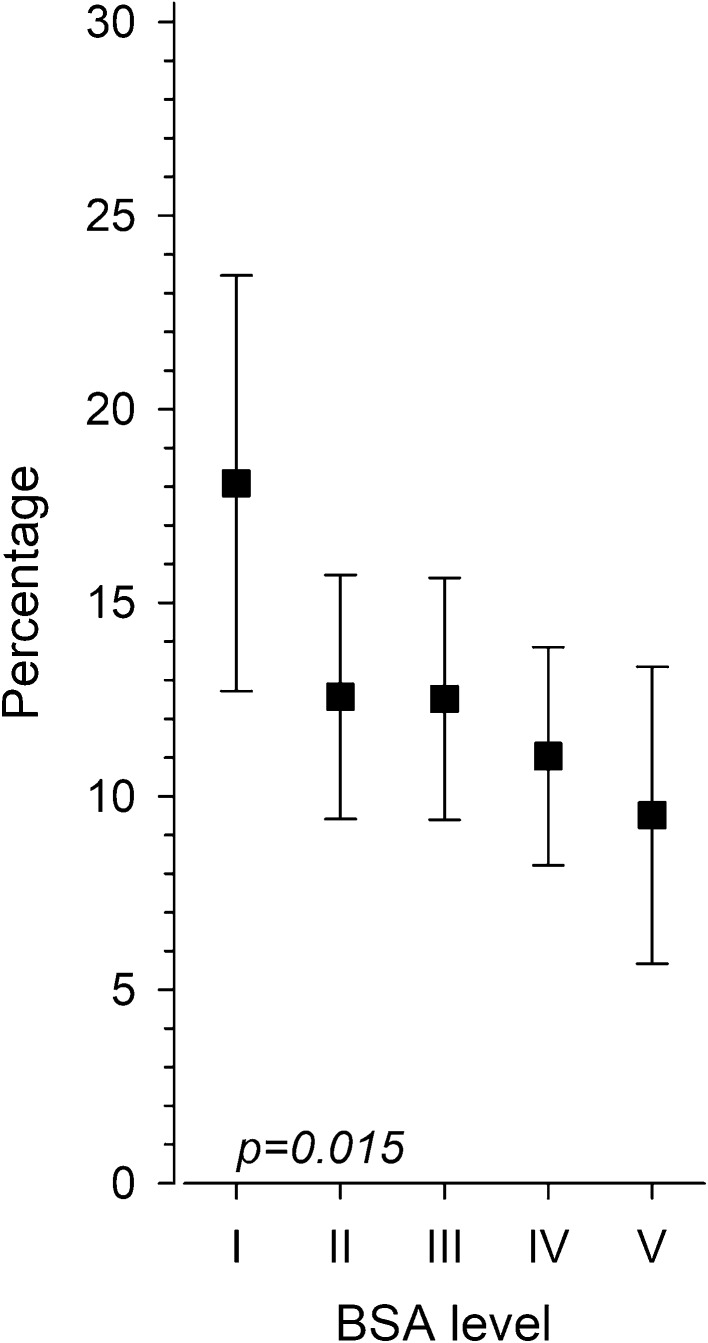
Prevalence of gestational diabetes mellitus (GDM) according to maternal body surface area (BSA) level at birth. Prevalence of GDM in percentages (%), according to five different maternal BSA levels at birth with the following cut-offs: 2011 cm^2^ ≤ for level I, 2012–2170 cm^2^ for level II, 2171–2291 cm^2^ for level III, 2292–2450 cm^2^ for level IV, and ≥ 2451 cm^2^ for level V. *p* value is calculated for linearity across the different levels after adjustments for age, educational attainment, pre-pregnancy body mass index, and smoking

**Fig. 3:**
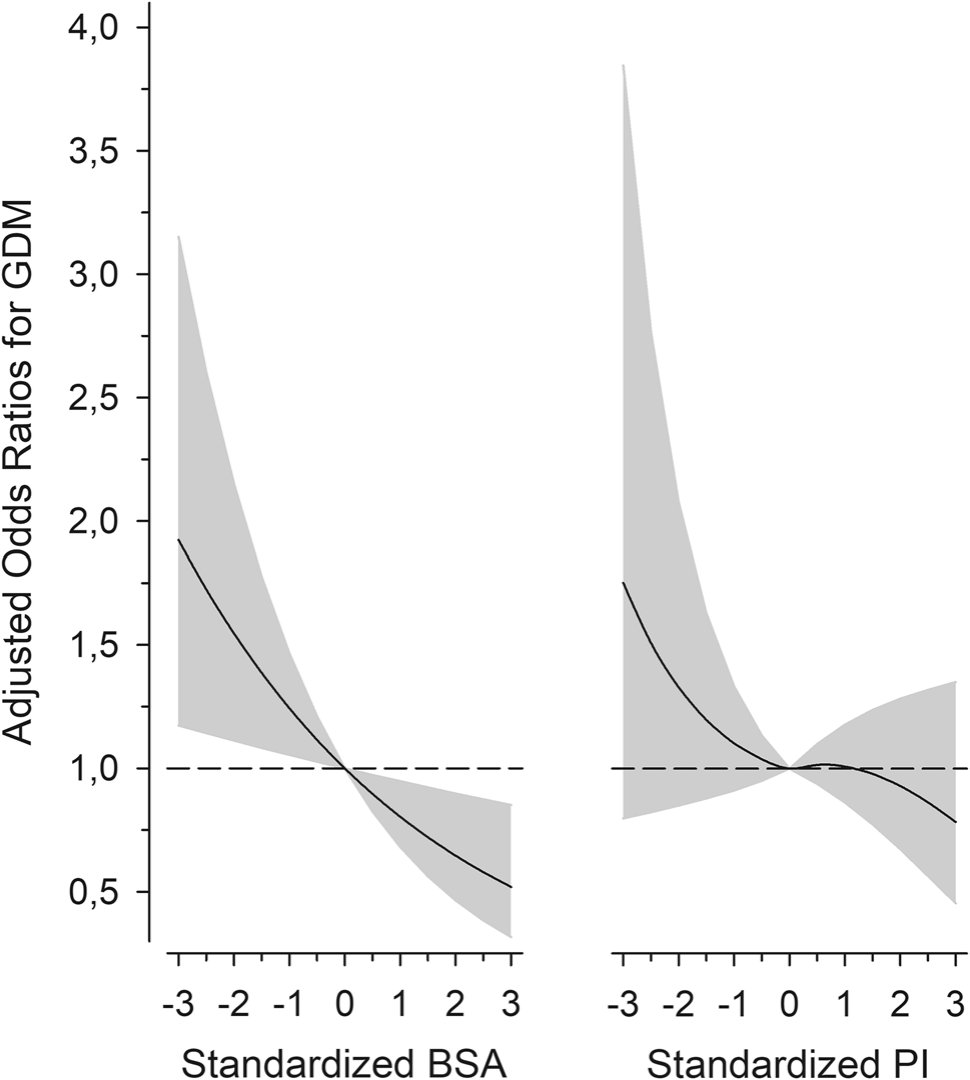
Risk for gestational diabetes mellitus (GDM) according to standardized maternal body surface area (BSA) at birth and to standardized maternal ponderal index (PI) at birth. Adjusted odds ratios for GDM according to maternal BSA at birth and to maternal ponderal index at birth, as standardized values on a continuous scale after adjustments for age, educational attainment, pre-pregnancy BMI, and smoking. Reference values are the average values of BSA and PI in the study cohort (standardized *Z*-score values at 0). The curves were derived from 5-knot-restricted cubic spline logistic models. Gray areas and whiskers represent 95% confidence intervals. *BMI* body mass index

Overall GDM prevalence was 12.3% (95% CI 2.3–14.0). BSA at birth was inversely associated with GDM (*p* = 0.015 for linearity, after adjustments for age, educational attainment, pre-pregnancy BMI and smoking). The highest prevalence of GDM, 18.1% (95% CI 12,7–23,5) was observed at level I; whereas, the lowest prevalence of GDM, 9.5% was observed at level V (95% CI 5,7–13,3) (Fig. 2). The odds ratio (OR) for GDM for those with the largest BSA (level V) compared with those with the smallest BSA (level I) was 0.43 (95% CI 0.22–0.83) after adjustments for the same confounders (Fig. 2). Figure 3 illustrates the continuous relationship between BSA at birth, PI at birth and risk for GDM. The OR for GDM was 0.80 (95% CI 0.68–0.95, *p* = 0.009) for each one SD increase in BSA at birth, and the OR for GDM was 0.95 (95% CI 0.80–1.12, *p* = 0.53) for each one SD increase in ponderal index at birth, after adjustment for the same confounders (Fig. 3).

## Discussion

According to our study findings, there is a linear inverse association between BSA at birth, and later risk for GDM in primiparous women. The prevalence of GDM was highest (18.1%), among those with the smallest BSA at birth, and lowest (9.5%), among those with the largest BSA at birth. Similarly, the OR for GDM in the group with the largest BSA, compared to the group with the smallest BSA was 0.46. We also used another marker of body size at birth, i.e., ponderal index to evaluate the relationship between body size at birth and risk for GDM. Ponderal index at birth did not predict future risk of GDM. Further, BSA at birth and adult anthropometry correlated, although weakly, with the strongest correlation observed between BSA at birth and adult height.

The GDM prevalence in our study cohort was 12% and the mean age of the primiparous women was 22 years. The mean age of primiparas in Finland during the study period between 2010 and 2015 was 29 years [28]. The nationwide prevalence of GDM in Finland during 2016 was 18% [28], which is higher than the prevalence in the present study. This is probably explained by the fact that the women in our study cohort were primiparas of rather young age, due to restrictions of our register-based study setting as the data is limited to women born after 1987, when the Finnish Medical Birth Register was founded.

To the best of our knowledge, this is the first study to investigate the relationship between BSA at birth and later risk for GDM in primiparous women. We aimed to investigate the relationship using BSA at birth as a more accurate estimate for body size at birth than for example birth weight, in order to approximate the metabolic tissue and body size as a whole. Further, we assessed the relationship between both BSA and ponderal index as parameters on risk for GDM. Comparisons between our findings and previous publications are based on studies that have assessed the relationship between birth weight only or ponderal index, and risk for GDM.

Similar to previous studies that have shown birth weight to be inversely associated with risk for GDM [16–18, 29, 30], and a more recent study from 2017 that reported ponderal index to be inversely associated with GDM [22], we also found an increased risk for GDM in women with a small BSA at birth and a lower risk for women with a large BSA at birth. However, we did not detect a similar statistically significant relationship between ponderal index and risk for GDM. Further, we did not detect a U-shaped relationship between small and large infants and risk for GDM, as some previous studies have reported with respect to low and high birth weight and risk for GDM [19–21, 31, 32].

The conflicting results between former studies with regard to a linear inverse versus a U-shaped relationship between birth weight and risk for GDM have, at least to some extent, been thought to reflect differences in study settings, with some studies lacking a big enough comparison group of macrosomic infants. In addition, the relationship has also been explained by ethnic differences; as Williams showed in 1999, women of African-American ethnicity showed a U-shaped relationship between birth weight and risk for GDM, while women of other ethnicities, showed an inverse linear relationship [33]. Moreover, offspring born to pregnant women who have been diagnosed with GDM are more prone to be macrosomic, and maternal GDM has been recognized as a risk factor for future metabolic disturbances in the offspring [5].

There are several factors affecting fetal growth such as gestational age, parity, infant sex, in utero metabolism, and genetic factors [34, 35]. Furthermore, a small body size at birth can be explained by malnutrition during pregnancy [36], or maternal constraints [37], due to limited space in utero as a result of a narrow pelvis.

A small birthweight has been thought to affect morbidity in adulthood and has formed the basis for the DOHaD hypothesis [38]—the concept that the in utero environment, developmental plasticity, and possible epigenetic mechanisms during critical periods of early organ development can have long-lasting effects on health [38]. Moreover, low birth weight has been linked to insulin resistance [39]. In 1991, Hales and Barker proposed in their Hertfordshire study that infants born small have an impaired glucose tolerance in adulthood and that this might be due to impaired development of the endocrine pancreas and result in impaired beta cell function later in life [40]. Further, insulin is recognized to be an important growth factor and studies indicate that there could be genetic alleles that might both reduce fetal growth and cause an impaired insulin secretion and hence, predispose to diabetes [41].

Why did we think it was important to assess the relationship between maternal body size as a whole, also taking birth length into account, and risk for GDM? Compared to birth weight, birth length has been considered to be an even stronger predictor of adult height [42, 43], and height has been shown to influence the risk for GDM [10].

In 2005, Eide et al. concluded that as birth length predicts adult stature and adult stature is associated with several non-communicable disorders—birth length might be a better predictor of adult health than birth weight [42]. Adult weight is to a greater degree influenced by environmental factors and appears to have a weaker hereditary component than height [42]. Therefore, in order to have a more accurate measurement of maternal body size at birth, taking both birth weight and birth length into account and to further dilute the effect of birth weight as the only measurement of birth size in assessing the risk of disease burden in adulthood, we wanted to evaluate the risk for GDM using both BSA and ponderal index at birth as measurements. According to our results, BSA has a stronger effect on predicting risk for GDM than ponderal index has. Our findings suggest that risk for GDM is inversely associated with BSA at birth. Ponderal index at birth showed no significant effect on risk for GDM.

The strength of the study is that it encompasses all primiparous Finnish women from Vantaa city, the fourth biggest city in Finland, who delivered during a 7-year follow-up period and of whom we had complete data about their own birth length and weight based on register data. To avoid the confounding effect of previous GDM or parity, we included only primiparas in the study. The diagnosis of GDM is based on a standardized 2-h 75-g OGTT and the diagnostic criteria have remained the same during the whole study period. Finally, the Finnish Medical Birth Register is considered to be of high quality [44].

The study also has some limitations. We missed information about some well-known risk factors for GDM; such as family history of diabetes, gestational weight gain, dietary habits, and physical activity. All study participants had Finnish background, and therefore the generalization of the results globally can be restricted. Most importantly, it was only in 1987 when the Finnish Medical Birth Register started to collect information on a nationwide basis, thereby, this cohort consisted only of rather young primiparas and the results cannot be generalized to older pregnant women.

In conclusion, BSA at birth is inversely associated with future risk for GDM in primiparous women. Special attention should be paid to pregnant women who have been born small, in order to follow-up and if possible reduce the risk for GDM. Likewise, to encourage a healthy and nutritious pregnancy diet and to aim for an optimal fetal growth are important in any pregnancy to reduce transgenerational transmission of GDM and to prevent an infant’s risk for being born small and thereby at risk for future metabolic disturbances.
